# Inflammation-Based Cell Ratios Beyond White Blood Cell Count for Predicting Postimplantation Syndrome After EVAR and TEVAR

**DOI:** 10.3390/ijms26199753

**Published:** 2025-10-07

**Authors:** Ebubekir Sönmez, İzatullah Jalalzai, Ümit Arslan

**Affiliations:** 1 Department of Cardiovascular Surgery, Kütahya City Hospital, Kütahya 43100, Türkiye; e.sonmezkvc@gmail.com; 2 Department of Cardiovascular Surgery, Faculty of Medicine, Atatürk University, Erzurum 25030, Türkiye; ijalalzai@gmail.com

**Keywords:** postimplantation syndrome, endovascular aneurysm repair, EVAR, thoracic endovascular aortic repair, TEVAR, neutrophil-to-lymphocyte ratio, systemic inflammatory response index, eosinophil-to-lymphocyte ratio, C-reactive protein, albumin

## Abstract

Postimplantation syndrome (PIS) is an early inflammatory response following endovascular stent-graft implantation (EVAR and TEVAR), defined by culture-negative fever and leukocytosis. The patient’s preoperative inflammatory status is thought to play a central role in its development. This study aimed to evaluate whether the systemic inflammatory response index (SIRI) and the eosinophil-to-lymphocyte ratio (ELR) can serve as preoperative predictors of PIS. Clinical data from 300 patients who underwent aortic endograft implantation and laboratory results obtained 24 h before the procedure, and at 24 h, 72 h, and 1 week postoperatively, were prospectively recorded. PIS was defined as culture-negative fever ≥ 37.8 °C accompanied by leukocytosis ≥ 12,000/µL. Inflammation-based indices derived from complete blood count (SIRI and ELR), along with serum C-reactive protein (CRP) and albumin levels, were compared between patients with and without PIS. Logistic regression and receiver operating characteristic (ROC) analyses were performed to identify independent predictors. PIS developed in 55 patients (18.3%). Patients with PIS were younger (70.1 ± 8.6 vs. 72.7 ± 7.3 years; *p* = 0.042) and had larger aneurysm diameters and greater mural thrombus thickness. Preoperatively, leukocyte count, SIRI, and CRP levels were significantly higher in patients who developed PIS, whereas ELR and albumin levels were lower. Multivariable analysis showed that a larger aneurysm diameter (OR: 1.2; 95% CI: 1.0–1.3; *p* = 0.003), greater mural thrombus thickness (OR: 1.3; 95% CI: 1.0–1.6; *p* = 0.012), EVAR procedure (OR: 3.7; 95% CI: 1.2–6.3; *p* = 0.033), elevated SIRI (OR: 1.9; 95% CI: 1.2–3.1; *p* = 0.005), and higher CRP (OR: 1.4; 95% CI: 1.1–3.2; *p* = 0.003) were significantly associated with PIS. In contrast, increasing age, higher ELR, and higher albumin levels were associated with a reduced risk of PIS. Simple biomarkers routinely obtained from standard laboratory tests can contribute meaningfully to the preoperative prediction and postoperative identification of PIS. Their integration into risk stratification models and confirmation against definitive diagnostic criteria will require validation in larger, multicenter studies.

## 1. Introduction

Endovascular repair of aortic aneurysms, including both EVAR and TEVAR, has become the first-line treatment because of its low perioperative mortality and clear clinical advantages over open repair [1]. Regardless of the technique used, the interaction between the implanted graft and the host’s cellular and biochemical responses consistently leads to systemic inflammation [2]. Galle et al. [3] reported that, compared with open surgery, the endoluminal approach provokes a weaker acute-phase response, less T-lymphocyte activation, and reduced complement release. Schürmann et al. [4] experimentally confirmed the presence of this acute-phase response, and Velázquez et al. [5] later described postimplantation syndrome (PIS) and emphasized its clinical importance.

PIS is generally defined as a postoperative condition characterized by fever, leukocytosis, elevated C-reactive protein (CRP), and fatigue in the absence of infection. However, the absence of a uniform definition has led to wide variation in its reported incidence [6,7,8]. Aytekin and Iscan [9] noted that patients often described their symptoms as “flu-like,” which may contribute to its frequent underestimation in clinical practice. Inflammation, nonetheless, appears to be central to the pathogenesis of more complicated postoperative courses [10]. In patients who develop PIS, studies have shown higher rates of major adverse cardiovascular events within the first year, prolonged hospital stay, and increased readmission, all of which are linked to an exaggerated inflammatory response [11,12,13,14,15,16]. Although the condition may initially present with mild or nonspecific findings, its potential to progress to serious complications highlights the need for a standardized diagnostic framework [17].

The most frequently investigated biomarkers for the early identification of PIS include CRP, leukocyte count, and interleukin-6 (IL-6). However, the prognostic significance and clinical applicability of inflammation-based indices derived from routine complete blood count remain insufficiently defined [18]. Recent studies have demonstrated that elevated neutrophil-to-lymphocyte ratio (NLR) and systemic inflammatory response index (SIRI) are associated with worse short- and long-term outcomes after endovascular aortic repair [19,20,21]. By contrast, the role of eosinophils—important mediators in the inflammatory cascade—has received comparatively little attention [22]. Low eosinophil count has been linked to poor prognosis in aortic disease [23,24], and Bass [25] reported that acute inflammatory triggers frequently result in eosinopenia. More recently, the eosinophil-to-lymphocyte ratio (ELR) has been proposed as a promising marker of inflammatory burden [26].

The aim of this prospective study was to evaluate the predictive value of SIRI—which incorporates the NLR—and ELR, as well as to assess whether preoperative CRP and albumin provide additional prognostic information in patients undergoing endovascular aortic repair.

## 2. Results

A total of 300 patients were included: 170 underwent EVAR (29 female, 141 male) and 130 underwent TEVAR (35 female, 95 male). The mean age was 72.2 ± 7.6 years. Hypertension was present in 85% of patients, chronic obstructive pulmonary disease (COPD) in 69%, and 32% had diabetes mellitus. Regarding smoking status, 44% were former and 36% current smokers. Coronary artery disease (CAD) was present in 62 patients, and 14 had previously undergone coronary artery bypass grafting (CABG). In addition, 15 patients had a history of cerebrovascular events, and 24 had peripheral artery disease (PAD).

As the primary outcome, PIS was identified in 40 patients (23.5%) of the EVAR group and 15 patients (11.5%) of the TEVAR group. Post hoc power analysis using G*Power (two-tailed with α = 0.05) demonstrated a power of 76% with an effect size of Cohen’s h = 0.35, indicating a study with moderate statistical power.

**Figure 1:**
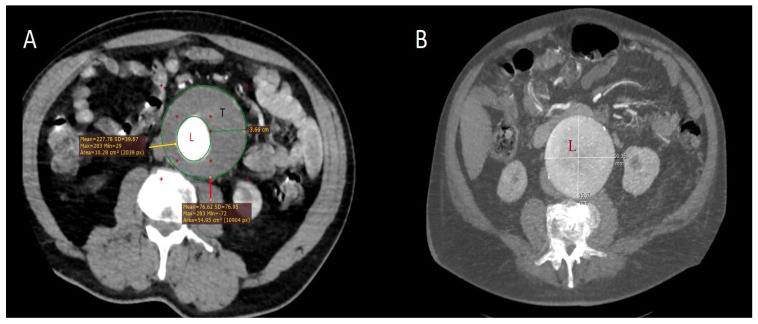
Computed tomography (CT) images illustrating different morphologic features of abdominal aortic aneurysms. (**A**) In a 69-year-old male patient who developed PIS, subtraction of the aortic lumen area (L; yellow arrow; red “+” symbols indicate the measurement boundaries in the analyzed area) from the total aneurysm area (red arrow) demonstrates a mural thrombus (T) with a maximum thickness of 3.66 cm in the transverse plane, corresponding to a cross-sectional area of at least 44 cm^2^. Although aneurysms are inherently three-dimensional structures, planar measurements provide clinically relevant information on thrombus burden. (**B**) In a 72-year-old female patient who developed PIS, an aneurysm measuring nearly 10 cm in maximum diameter was observed. The aortic lumen (L) was entirely encompassed by the aneurysmal sac, appearing uniformly dilated with smooth margins and without mural thrombus formation. At the 6-month follow-up, however, thrombosis of the excluded lumen following EVAR resulted in an apparent increase in aortic wall thickness. These contrasting examples suggest that aneurysm morphology—including thrombus presence, thickness, and sac size—may influence the risk of PIS.

Patients who developed PIS were younger (mean difference, 2.6 years; *p* = 0.042), and 42 were male. Within this group, 51 had hypertension, 39 had COPD, 39 had atherosclerotic cardiovascular disease, and 25 were current smokers. They also exhibited larger aneurysm transverse diameters and greater mural thrombus thickness (Figure 1A,B). Operative times were at least 20 min longer, resulting in greater use of contrast media. In addition, temperature in patients with PIS increased on postoperative day 2 (Q1–Q3: 1–3), reaching a median of 38.3 °C (Q1–Q3: 37.8–39.1; *p* < 0.001). Their hospital stay was also prolonged by approximately one week compared with patients without PIS [13 days (Q1–Q3: 11–14); *p* < 0.001].

Based on laboratory assessments, patients who developed PIS had lower preoperative hemoglobin, vitamin D, and albumin levels, whereas serum creatinine, LDL cholesterol levels were higher. Inflammatory profiles also differed: preoperative white blood cell (WBC), neutrophil, and monocyte count, together with CRP levels, were elevated, while lymphocyte and eosinophil counts were reduced. Consequently, preoperative NLR, SIRI, and ELR values showed significant differences between patients with and without PIS. A detailed comparison of clinical characteristics and preoperative laboratory findings is presented in Table 1.

To identify predictors of PIS, a multivariable logistic regression model was constructed, incorporating age, sex, aneurysm diameter, intraluminal thrombus thickness, and procedure type as a priori variables. Preoperative laboratory parameters included albumin, CRP, ELR, and SIRI. To minimize multicollinearity among indices derived from overlapping cell counts, only SIRI was retained, supported by an acceptable variance inflation factor (VIF = 1.1) and its integrative value reflecting both neutrophil and monocyte counts. The model demonstrated strong discriminative ability (AUC = 0.955), with 94% specificity and 78% sensitivity. In multivariable analysis, EVAR (OR: 3.7), younger age (≤69.5 years), larger aneurysm diameter (>65 mm), greater thrombus thickness (>21.5 mm), elevated CRP (>8.7 mg/L), elevated SIRI (≥2.2), reduced ELR (<0.08), and reduced albumin (<3.8 g/dL) were identified as independent predictors of PIS, as summarized in Table 2.

**Figure 2:**
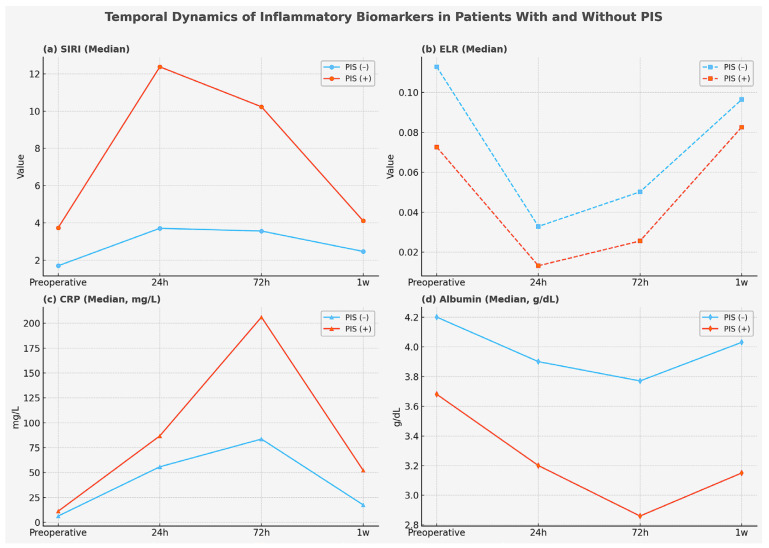
Temporal dynamics of inflammatory biomarkers in patients with and without postimplantation syndrome (PIS). Median values of systemic inflammatory response index (SIRI, panel (**a**)), eosinophil-to-lymphocyte ratio (ELR, panel (**b**)), C-reactive protein (CRP, mg/L; panel (**c**)), and albumin (g/dL; panel (**d**)) are shown at baseline (preoperative), 24 h, 72 h, and 1 week postoperatively. Patients with PIS exhibited markedly higher SIRI and CRP values, persistently lower ELR, and reduced albumin concentrations compared with those without PIS, particularly in the early postoperative period.

These biomarkers were also evaluated in the postoperative period. Patients with PIS exhibited consistently higher NLR values, SIRI values, and CRP levels across all three postoperative time points (all *p* < 0.001, Brunner–Munzel test). ELR reached its lowest levels at 24 h, corresponding to a marked decline in eosinophil counts (*p* < 0.001). By the first postoperative week, however, the inflammatory response appeared to subside, with eosinophil counts rising to a median of 120 cells/μL, and the difference in ELR between groups was no longer statistically significant (*p* = 0.108). The temporal evolution of inflammatory biomarkers (SIRI, ELR, CRP, and albumin) in patients with and without PIS is illustrated in Figure 2a–d.

Procedural duration was significantly associated with PIS, with each additional 10 min of operative time increasing the risk by approximately 20% (OR: 1.20; 95% CI: 1.11–2.08; *p* < 0.001). In addition, longer operative duration was positively correlated with postoperative SIRI values at both 24 h (Spearman’s ρ = 0.452, *p* < 0.001) and 72 h (ρ = 0.302, *p* < 0.001). Significant positive correlations were also observed with CRP at 24 h (ρ = 0.313, *p* = 0.002) and 72 h (ρ = 0.455, *p* < 0.001). By contrast, ELR values showed inverse correlations, being significantly reduced at 24 h (ρ = −0.474, *p* = 0.013), whereas the negative correlation at 72 h did not reach statistical significance (ρ = −0.241, *p* = 0.071).

Postoperative ROC analyses were performed to examine the adjunctive diagnostic role of inflammation-based formulas and biochemical markers in patients with PIS. At 24 h, the optimal cut-off values were SIRI ≥ 6.1 (AUC: 0.966), ELR ≤ 0.02 (AUC: 0.660), CRP ≥ 70.5 mg/L (AUC: 0.880), and albumin ≤ 3.4 g/dL (AUC: 0.851). At 72 h, the corresponding thresholds were SIRI ≥ 6.0 (AUC: 0.924), ELR ≤ 0.05 (AUC: 0.704), CRP ≥ 160 mg/L (AUC: 0.980), and albumin ≤ 3.2 g/dL (AUC: 0.885). Table 3 summarizes the diagnostic performance of markers for PIS. Among these, CRP and SIRI provided the strongest support for the diagnosis, while ELR contributed only modestly. Albumin, although traditionally regarded as a marker of nutritional status, also demonstrated predictive value in the context of systemic stress and inflammation, highlighting the interplay between metabolic reserve and the inflammatory response in patients with PIS.

## 3. Discussion

This prospective study demonstrated that inflammation-based indices derived from leukocyte subgroups, specifically SIRI and ELR, may serve as supportive markers not only for the preoperative prediction of PIS but also for assisting its recognition in the postoperative setting, particularly when leukocytosis and fever are present while culture results are still pending. We found that patients younger than 70 years, scheduled for EVAR, with larger aneurysm diameters, greater intramural thrombus thickness, elevated preoperative SIRI, and reduced ELR could be more accurately identified as being at risk when CRP and albumin levels were incorporated into the model. Using these variables, the model correctly classified 78% of patients with PIS. This level of predictive accuracy suggests that closer perioperative vigilance, careful limitation of contrast media use, and structured postoperative monitoring may represent practical measures to mitigate risk and improve outcomes in this population.

Over the past three decades, endovascular stent-graft implantation has been widely adopted worldwide because of its multiple advantages over open surgery [27]. These benefits are attributed not only to its minimally invasive nature but also to the attenuated inflammatory response compared with the pronounced systemic activation typically triggered by open repair [28]. Early studies indicated that endovascular interventions evoke a less intense acute-phase reaction, characterized by reduced T-lymphocyte activation and complement activity, which may contribute to a lower incidence of multiple organ dysfunction [29,30]. Nevertheless, Blum et al. [7] reported that nearly half of 154 patients developed fever, leukocytosis, and elevated CRP lasting 4–10 days, despite negative blood cultures and the absence of graft infection. Velázquez et al. [5] subsequently introduced this constellation of findings into the literature as PIS.

Although PIS is generally regarded as a clinical manifestation within the spectrum of systemic inflammatory response syndrome (SIRS), the absence of universally accepted diagnostic criteria renders its true incidence uncertain and increases the risk of misclassification or underdiagnosis [31,32]. Classically, PIS emerges 2–3 days after the procedure and is characterized by persistent fever despite antibiotic therapy, negative culture results, and leukocytosis. However, no uniform leukocyte cut-off value has been established, and some investigators have proposed that CRP elevation should also be incorporated into the definition [33,34,35,36]. In our study, PIS was defined by negative cultures, body temperature ≥ 37.8 °C, and leukocyte count ≥ 12,000/μL [9,37]. Using these criteria, the incidence of PIS was 23.5% (n = 40) after EVAR and 11.5% (n = 15) after TEVAR.

According to our institutional protocol, cultures are routinely obtained in all patients with postoperative fever, and in those who ultimately fulfilled PIS criteria in this study, culture results were consistently negative. A major challenge, however, lies in the reliance on microbiological confirmation, as culture results typically require 24–72 h. During this waiting period, fever and leukocytosis cannot be formally classified as PIS, which may delay recognition and contribute to underreporting. The absence of standardized timing for culture collection across centers introduces further variability. These limitations likely explain part of the heterogeneity in reported PIS incidence and underscore the need for uniform diagnostic criteria in future studies.

The pathophysiology of PIS remains incompletely understood; however, its inflammatory triggers are clearly multifactorial, encompassing patient demographics, aneurysm morphology, stent-graft material, contrast media exposure, and procedural techniques [38,39,40,41]. In our cohort, patients who developed PIS displayed a more adverse comorbidity profile, including hypertension, COPD, CAD, elevated creatinine without dialysis, and active smoking. They also demonstrated unfavorable anatomical and procedural characteristics, such as larger aneurysm diameters and greater thrombus thickness, which in turn necessitated longer operative times and greater volumes of contrast media. Collectively, these factors may have amplified the inflammatory response observed in this subgroup.

The clinical importance of identifying such predictors lies in the heterogeneous course of PIS, which may range from a transient flu-like syndrome to significant morbidity and even mortality [42]. A meta-analysis reported significantly higher rates of 30-day mortality (0.6% vs. 0%; *p* = 0.03) and major adverse cardiac events (5.8% vs. 0.43%; *p* < 0.0001) in patients with PIS [43]. Consistent with these findings, Arnaoutoglou et al. [13] emphasized the potential for disease progression and advocated vigilant monitoring throughout the first postoperative month. In contrast, Chatzelas et al. [44] observed no 30-day mortality, and in the meta-analysis by Oztürk et al. [45], mortality did not differ significantly between patients with and without PIS (OR: 0.27; 95% CI: –0.27 to 0.81; *p* = 0.33). Despite such variability, our experience confirms that PIS should not be underestimated: while negative blood cultures may provide reassurance, the prolonged hospitalization, heightened patient and family anxiety, and systemic consequences of severe inflammation make the description by Reyes [46]—“not good, but not bad either”—strikingly appropriate.

As endovascular therapy has become the preferred treatment for nearly all forms of aortic aneurysm [47], the number of reported PIS cases is also expected to rise. The underlying mechanism likely reflects an inflammatory process already present within the aneurysm sac that is further amplified by stent-graft deployment [3,30,48,49,50,51,52]. Most prior investigations have concentrated on the postoperative phase, aiming to characterize this response and its clinical consequences [38,53,54]. These studies have highlighted several diagnostic markers, including leukocyte count, high-sensitivity CRP (hs-CRP), IL-6, IL-8, TNF-α, and other cytokines [2,12,36,48,55]. However, widespread application of these markers is hampered by limited assay availability, as test kits are not routinely accessible in many hospitals. In addition, the inflammatory cascade is inherently complex and may be influenced by multiple perioperative factors—such as allergic reactions, anesthesia, and surgical trauma—making it difficult to attribute postoperative biomarker elevations solely to PIS [56,57,58].

Given the established role of inflammation in aortic disease, the development of PIS may be anticipated by integrating demographic factors with preoperative laboratory profiles [59,60]. Immunoinflammatory mechanisms implicated in aneurysm pathophysiology—such as immune cell activation and IL-6 release from mural thrombus—likely overlap with those driving PIS, while surgical induction of acute-phase reactants further amplifies this response [61,62,63]. Supporting this concept, the ARIC Study [64] showed that abnormal differential leukocyte counts in midlife were significantly associated with future abdominal aortic aneurysm, with the strongest risk observed for above-normal neutrophil counts (HR 2.17; 95% CI, 1.29–3.64). Notably, even below-normal neutrophil, lymphocyte, eosinophil, and basophil counts were linked to increased risk (HRs 1.62–1.86). These findings suggest that formulas derived from standard preoperative complete blood counts may serve as practical tools to assess inflammatory status and anticipate the likelihood of PIS.

Although cut-off values vary across studies, WBC count remains an important criterion in the diagnosis of PIS [17]. Its consideration in the preoperative period may also have predictive value. Leone et al. [65] reported that preoperative WBC count was a significant predictor of PIS (OR 1.1, *p* = 0.047). In our cohort, patients who developed PIS likewise exhibited higher preoperative WBC count (*p* < 0.001), consistent with the findings of Leone et al. [65]. However, WBC count alone may not adequately reflect the complexity of the inflammatory response. Indices derived from cellular interactions or combined ratios may provide a more informative assessment [57]. Composite markers such as the systemic immune-inflammation index (SII), systemic inflammation response index (SIRI), aggregate index of systemic inflammation (AISI), platelet-to-lymphocyte ratio (PLR), neutrophil-to-lymphocyte ratio (NLR), eosinophil-to-lymphocyte ratio (ELR), and monocyte-to-lymphocyte ratio (MLR) are particularly appealing, as they can be easily calculated from routine complete blood counts and represent practical measures of systemic inflammatory burden [66,67].

In our cohort, NLR, SIRI, and ELR values were calculated; however, because SIRI already incorporates both neutrophil and lymphocyte counts, only SIRI was retained in the regression model to avoid multicollinearity. This decision was supported by variance inflation factor (VIF) analysis, which confirmed acceptable collinearity and indicated that SIRI provided more robust clinical insights [68]. Importantly, SIRI also integrates monocyte count, a cell type highlighted by Klopf et al. [69] as playing a pivotal role in aortic aneurysm biology and in coordinating inflammatory cascades. In our regression model, a preoperative SIRI above 2.2 emerged as an independent predictor of PIS (*p* = 0.005), conferring nearly a twofold increased risk (OR 1.9; 95% CI, 1.2–3.1). Furthermore, in the postoperative setting, SIRI values ≥ 6.1 at 24 h (AUC: 0.966) and ≥6.0 at 72 h (AUC: 0.924) supported the diagnosis of PIS. These elevations were driven by shifts across all three cellular components, but were predominantly influenced by neutrophil increases.

Even when considered independently, NLR retained predictive utility. A preoperative NLR ≥ 3.5 predicted PIS with good accuracy (AUC: 0.892), while postoperative values ≥ 8.5 at 24 h and ≥7.8 at 72 h also supported the diagnosis. Taken together, these findings underscore that the interplay of elevated neutrophils and reduced lymphocytes identifies patients more vulnerable to PIS, consistent with the observations of Octeau et al. [20]. Therefore, as emphasized in prior studies, detailed assessment of inflammation-based cell ratios is essential for reliable risk stratification and prognostication in this patient population [70,71,72].

Eosinophils are multifunctional leukocytes with pleiotropic effects that not only initiate and amplify inflammatory responses but also play a central role in maintaining immune homeostasis. By bridging innate and adaptive immunity, they exert regulatory functions that extend beyond their classical proinflammatory activity [73,74]. Experimental data even suggest a protective role of properly functioning eosinophils against aortic aneurysm formation [75].

In the surgical setting, however, stress-induced glucocorticoids such as cortisol can lead to eosinopenia [76]. Zhao et al. [23] reported that in patients undergoing endovascular repair for type B aortic dissection, eosinopenia was an independent risk factor for aorta-related mortality. Similarly, Von Meijenfeldt et al. [77] observed that in patients undergoing non-cardiac vascular surgery, lower eosinophil counts were associated with more severe acute inflammation, longer hospitalization, and a higher 90-day mortality risk (OR 1.97).

Grochowiecki et al. [78] further highlighted the prognostic relevance of eosinophils in patients after endovascular repair. Although no cases of PIS were identified in their cohort, eosinophil dynamics showed clinical importance: higher preoperative counts demonstrated a non-significant protective trend (HR 0.02; *p* = 0.18), while elevated eosinophils on the third postoperative day were independently associated with reduced aorta-related mortality (HR ≈ 1.9 × 10^−6^; *p* = 0.038). This effect persisted as a borderline trend by day 5 (HR 0.0004; *p* = 0.067).

In our study, eosinophil counts were consistently lower in patients with PIS at all time points, dropping below 40 cells/μL on postoperative days 1 and 3. By day 7, both eosinophil and lymphocyte count showed evidence of recovery, likely reflecting attenuation of the inflammatory response. In multivariate logistic regression, low preoperative ELR values (<0.08; AUC = 0.641) demonstrated only limited discriminative ability for identifying PIS, suggesting that this parameter should be interpreted alongside other risk factors. Postoperatively, however, ELR values ≤ 0.05 on day 3 supported the diagnosis of PIS with moderate accuracy (AUC = 0.704). These findings are consistent with the observations of Shah et al. [79], who underscored the adverse prognostic implications of eosinopenia.

## 4. Materials and Methods

### 4.1. Study Design and Patient Selection

The present prospective, single-center observational cohort study was conducted between January 2018 and December 2024, and the study protocol was registered at ClinicalTrials.gov (ClinicalTrials.gov Identifier: NCT07014839; U.S. National Library of Medicine—National Institutes of Health, Bethesda, MD, USA, 2024; https://clinicaltrials.gov/study/NCT07014839?term=NCT07014839&rank=1, accessed on 6 October 2025). A total of 368 patients were evaluated for eligibility, of whom 170 undergoing EVAR and 130 undergoing TEVAR met the predefined inclusion criteria and were enrolled. Demographic characteristics, comorbidities, perioperative management, and treatments administered during intensive care unit and ward stays were systematically documented.

The maximum transverse diameters of the aortic aneurysm, along with the greatest mural thrombus thickness, were measured by computed tomography (CT). All measurements were performed manually and in a blinded manner by cardiovascular surgeons with more than ten years of professional experience, using standardized imaging protocols. Analyses were conducted both on centerline reconstructions generated with 3D Slicer software (v5.8; Harvard University, Cambridge, MA, USA) and on axial images using RadiAnt DICOM Viewer (v4.6.9; Medixant, Poznań, Poland).

Given the dynamic nature of the inflammatory response [48], venous blood samples were collected at four predefined time points: preoperatively (within 24 h before the procedure), and postoperatively at 24 h, 72 h, and one week. All samples were collected and documented by the same investigators (ES and IJ). In addition, the type of procedure, operative duration (time from skin incision to closure), and contrast volume (iohexol 300 mg/mL) were recorded.

During intensive care and ward follow-up, hemodynamic parameters and tympanic temperature were measured by bedside nurses blinded to the study objectives, and records were reviewed daily by the investigators. If body temperature reached or exceeded 37.7 °C, blood, urine, and throat cultures were obtained, and patients were monitored closely for fever. Patients were classified as having PIS if, within the first five postoperative days, they demonstrated leukocytosis (WBC ≥ 12 × 10^3^/μL) and body temperature ≥ 37.8 °C in the absence of positive microbiological cultures [9,37,80].

Based on blood sample analyses, the NLR was calculated as the ratio of absolute neutrophil count to absolute lymphocyte count. The SIRI was calculated as (absolute neutrophil count × absolute monocyte count)/absolute lymphocyte count. The ELR was calculated as the ratio of absolute eosinophil count to absolute lymphocyte count [26,81,82]. All clinical and laboratory variables were then compared between patients with and without PIS.

### 4.2. Exclusion Criteria

Exclusion criteria were as follows: ruptured aneurysm (n = 22), acute type B aortic dissection (n = 13), and traumatic injury (n = 3); procedures requiring left subclavian or femoral bypass (n = 6); active malignancy (n = 5); vasculitis (n = 2); and end-stage renal disease requiring dialysis (n = 3). Patients who died within the first postoperative week (n = 8) were also excluded to avoid incomplete serial inflammatory assessments. In addition, patients presenting with positive microbiological cultures together with a procalcitonin concentration > 0.5 ng/mL (n = 6) were excluded to minimize potential confounding from active infection [83]. After applying these criteria, 300 patients—170 treated with EVAR and 130 with TEVAR—were included in the final analysis

### 4.3. Technique

All procedures were performed in a hybrid operating suite equipped with a high-resolution C-arm fluoroscopy system. Anesthetic management consisted of either local anesthesia with sedation or general anesthesia, determined by institutional practice and patient-specific considerations. For EVAR procedures, bilateral surgical exposure of the common femoral arteries was undertaken, whereas TEVAR generally required only unilateral femoral access, depending on device configuration and anatomical feasibility. Intravenous heparin was administered intraoperatively to maintain an activated clotting time (ACT) above 180 s. Without repositioning the patient or operating table, angiographic localization of key branch vessels—including the subclavian and renal arteries—was carried out. The primary stent-graft (Lifetech Ankura endograft, Shenzhen, China), composed of expanded polytetrafluoroethylene (e-PTFE), was then deployed under controlled hypotension. Each procedure was completed with angiographic confirmation of technical success, defined as accurate endograft placement and the absence of endoleak.

### 4.4. Statistical Analysis

All statistical analyses were performed using SPSS (v28; IBM Corp., Armonk, NY, USA), Jamovi (v2.6.44; The Jamovi Project, Sydney, Australia), and G*Power (v3.1.9.7; Heinrich Heine University, Düsseldorf, Germany). Categorical variables were expressed as frequencies and percentages, and continuous variables as mean ± SD or median (IQR). Normality was tested with the Kolmogorov–Smirnov and Shapiro–Wilk tests, and variance homogeneity with Levene’s test. Group comparisons were performed using the chi-square or Fisher’s exact test for categorical data, and the independent-samples *t*-test, Mann–Whitney U test, or Brunner–Munzel test for continuous data, as appropriate.

Inflammatory indices (NLR, SIRI, ELR) across time points were analyzed using repeated-measures ANOVA or the Friedman test, with Bonferroni correction for multiple comparisons. ROC curve analysis was conducted to evaluate discriminatory performance, and the AUC with 95% CI was reported. To account for skewed distributions and small-sample bias, continuous variables were log-transformed (LN) before regression, and bias-corrected accelerated (BCa) bootstrapping with 5000 resamples was used to obtain robust 95% CIs. Independent predictors of PIS were first identified by univariate analyses (*p* < 0.10), followed by multivariable logistic regression. Odds ratios (ORs) with 95% CIs were calculated, and continuous predictors were standardized using Z-scores. A two-tailed *p* < 0.05 was considered statistically significant.

## 5. Limitations

Although this study was prospectively designed, its single-center nature may limit the generalizability of the findings to broader clinical settings. In addition, EVAR and TEVAR patients were analyzed as a combined cohort, which may have obscured procedure-specific differences. The diagnostic criteria used for PIS—negative cultures, fever, and leukocytosis—are widely applied but not universally standardized, and misclassification may therefore have occurred in some cases. As this investigation focused on preoperative prediction of PIS, mid- and long-term outcomes were not assessed, restricting the ability to fully determine the prognostic value of the biomarkers studied.

Furthermore, while we evaluated inflammatory cell ratios derived from simple leukocyte formulations, more detailed analyses of leukocyte subtypes are warranted, given that inflammation is a dynamic process shaped by multiple systemic and local factors. Another important limitation is the absence of cytokine measurements such as IL-6, IL-8, or TNF-α, which could have provided more mechanistic insight. In particular, IL-6 was not included due to the limited sample size available for this assay. Additionally, the role of intramural thrombus was only assessed by thickness rather than volumetric measurements, and potential effects of altered aneurysmal flow dynamics on inflammatory activation were not evaluated. Moreover, only a single type of stent-graft device was used in this cohort, precluding comparisons across different endograft platforms, which might also influence inflammatory responses. Finally, although preoperative albumin levels emerged as a significant predictor in our study, vitamin C and other micronutrients were not assessed, as they are not routinely measured in our clinic. This may represent an additional limitation, since micronutrient status could potentially influence both inflammatory activation and patient outcomes.

Taken together, these limitations highlight the need for larger, multicenter studies incorporating advanced biomarker and imaging analyses to refine risk stratification and better elucidate the pathophysiology of PIS.

## 6. Conclusions

This prospective study demonstrated that inflammation-based indices derived from routine blood counts, particularly SIRI and ELR, provide valuable information for both the preoperative prediction and postoperative recognition of PIS following endovascular aortic repair. Younger age, EVAR procedure, larger aneurysm size, greater thrombus thickness, elevated CRP and SIRI, lower ELR, and reduced albumin were identified as independent predictors. These findings support the role of simple, cost-effective cellular biomarkers in risk stratification, while highlighting the need for validation in larger multicenter cohorts and for the integration of specific inflammatory mediators. Modifiable factors such as anemia, dyslipidemia, vitamin D deficiency, and nutritional status may also deserve further exploration as potential targets for perioperative optimization. Finally, the lack of standardized diagnostic criteria underscores the necessity of a consensus definition of PIS to harmonize reporting, improve comparability, and guide clinical decision-making.

## Figures and Tables

**Table 1:** Demographic and Clinical Parameters in Patients with and without PIS.

Patient Demographics	Patients with PIS (n = 55)	Patients Without PIS (n = 245)	*p*
Female/male	13/42	51/194	0.645
Age (years)	70.1 ± 8.6	72.7 ± 7.3	0.042
EVAR/TEVAR, n (%)	40 (72.7)/15 (27.3)	130 (53.1)/115 (46.9)	0.010
Diabetes mellitus, n (%)	21 (38)	74 (30)	0.250
Hypertension, n (%)	51 (92)	203 (82)	0.066
Coronary artery disease, n (%)	20 (36)	42 (17)	0.002
Coronary artery bypass grafting, n (%)	5 (9)	9 (3.6)	0.110
Cerebrovascular event, n (%)	7 (12)	8 (3.2)	0.017
Peripheral artery disease, n (%)	7 (12)	17 (6.9)	0.121
COPD, n (%)	39 (70)	167 (68)	0.692
Current smoker, n (%)	25 (45)	83 (34)	0.134
Aneurysm diameter (transverse), mm, median (Q1–Q3)	77 (65–83)	65 (61–70)	<0.001
Intramural thrombus thickness, mm, median (Q1–Q3)	25 (21–28)	20 (16–25)	<0.001
Contrast volume, mL, mean	106	84	<0.001
Operative time, min, mean	110	90	<0.001
Hemoglobin, g/dL	13.5 ± 2.0	13.0 ± 1.8	0.574
^a^ White blood cell, 10^3^/μL, median (Q1–Q3)	10.4 (9.4–11.2)	8 (6.8–9.1)	<0.001
^a^ Neutrophil count, 10^3^/μL, median (Q1–Q3)	8.2 (7.2–8.7)	5.5 (4.5–6.8)	<0.001
^a^ Lymphocyte count, 10^3^/μL, median (Q1–Q3)	1.3 (1.1–1.8)	1.8 (1.5–2.0)	<0.001
^a^ Monocyte count, 10^3^/μL, median (Q1–Q3)	0.6 (0.5–0.9)	0.6 (0.5–0.7)	0.014
^a^ Eosinophil count, median (Q1–Q3)	0.1 (0.05–0.16)	0.2 (0.1–0.3)	<0.001
LDL, mg/dL	131 ± 28	110 ± 25	<0.001
HDL, mg/dL	41.1 ± 10.8	44.5 ± 12.7	0.093
Triglyceride, mg/dL, median (Q1–Q3)	125 (97–170)	112 (86–153)	0.141
Albumin, g/dL	3.6 ± 0.3	4.0 ± 0.4	<0.001
^a^ C-reactive protein, mg/L, median (Q1–Q3)	11 (9.0–13.2)	6.3 (5.0–8.2)	<0.001
Creatinine, mg/dL	1.0 ± 0.2	0.9 ± 0.1	0.039
HbA1c, %	6.1 ± 1.0	5.7 ± 0.8	0.122
Vitamin D level, ng/mL	12 ± 3.7	18 ± 6.3	<0.001
^b^ NLR; median (Q1–Q3)	5.6 (4.1–7.6)	3.0 (2.3–4.0)	<0.001
^b^ SIRI, median (Q1–Q3)	3.7 (2.5–5.5)	1.7 (1.3–2.2)	<0.001
^b^ ELR, median (Q1–Q3)	0.07 (0.03–0.13)	0.11 (0.07–0.15)	0.004

COPD, chronic obstructive pulmonary disease; ELR, eosinophil-to-lymphocyte ratio; HbA1c, Glycated hemoglobin; HDL, High-density lipoprotein; LDL, low-density lipoprotein; NLR, Neutrophil-to-lymphocyte ratio; PIS, postimplantation syndrome; SIRI, systemic inflammatory response index ^a^ Mann–Whitney U test; ^b^ Brunner–Munzel test.

**Table 2:** Multivariable logistic regression identifying preoperative predictors of PIS.

Predictor for PIS	OR (95% CI)	*p*
Sex (male)	1.2 (0.8–5.8)	0.869
Age, years	0.83 (0.73–0.95)	0.008
Aneurysm diameter, mm	1.2 (1.0–1.3)	0.003
Mural thrombus thickness, mm	1.3 (1.0–1.6)	0.012
Procedure type (EVAR vs. TEVAR)	3.7 (1.2–6.3)	0.033
Systemic inflammatory response index	1.9 (1.2–3.1)	0.005
Eosinophil-to-lymphocyte ratio	0.45 (0.2–0.9)	0.028
C-reactive protein, mg/L	1.4 (1.1–3.2)	0.003
Albumin, g/dL	0.63 (0.12–0.83)	0.013

**Table 3:** Diagnostic accuracy of inflammation-based indices and biochemical markers for postimplantation syndrome.

Time Point	Markers	Cut-off Values	AUC	Sensitivity (%)	Specificity (%)	Accuracy, %	PPV, %	NPV, %
At 24 h	SIRI	≥6.1	0.966	96.1	87.2	89.9	76.6	98.1
	ELR	≤0.02	0.660	74.5	56.8	62.1	42.7	83.8
	CRP, mg/L	≥70.5	0.880	90	76.1	79.3	54.5	95.1
	Albumin, g/dL	≤3.4	0.851	80	79.1	77.6	60.5	91.2
At 72 h	SIRI	≥6.0	0.924	90.2	85.1	86.7	73	95.1
	ELR	≤0.05	0.704	83.9	51.8	63.6	45.5	85.7
	CRP, mg/L	≥160	0.980	98.1	97.7	97.8	94.1	96.9
	Albumin, g/dL	≤3.2	0.885	91.1	78.2	81.2	62.2	83.6

AUC, Area under the curve; CRP, C-reactive protein; ELR, eosinophil-to-lymphocyte ratio; NPV, negative predictive value; PPV, positive predictive value; SIRI, systemic inflammatory response index.

## Data Availability

All data generated or analyzed during this study, including imaging materials, laboratory findings, and statistical data, are securely stored within the authors’ archives. No data were obtained from external sources or previously published materials. These datasets are available from the corresponding author upon reasonable request.

## Abbreviations

AUC	Area under the curve
CABG	Coronary artery bypass grafting
CAD	Coronary artery disease
COPD	Chronic obstructive pulmonary disease
CRP	C-reactive protein
ELR	Eosinophil-to-lymphocyte ratio
EVAR	Endovascular aneurysm repair
NLR	Neutrophil-to-lymphocyte ratio
OR	Odds ratio
PAD	Peripheral artery disease
PIS	Postimplantation syndrome
ROC	Receiver operating characteristic
SIRI	Systemic inflammatory response index
TEVAR	Thoracic endovascular aortic repair
